# Observations on Fœcal Obstruction

**Published:** 1853-12

**Authors:** Robert Christison


### *Observations on a case of Fœcal Obstruction.*

FROM A LECTURE ON CLINICAL MEDICINE, BY ROBERT CHRISTISON, M. D.

The occurrence of a singular case of obstruction of the intestines from accumulation of fæces, induces me to make a few remarks on a subject which, though it may appear trite to you, is really one of great importance, and deserving your early consideration as professional men.

When you enter presently on medical practice, you will probably be surprised, as I was in the same conjecture, at the exceeding frequency of the habit of constipation among people of easy circumstances in this country. At what period this liability was observed, and in what cause or causes it originates, are questions which at present I cannot pretend to discuss. But there can be no doubt of the fact, that the infirmity of constipation is extremely common; and likewise, that it often exists without any other constitutional infirmity or special disease, except what is clearly referable to an undue neglect of the proper correctives. Accordingly, by due attention to the use of fit laxatives, thousands of persons of both sexes in the middle and upper walks of life contrive to live as long, as healthy, and, except for the plague of constantly taking physic, as happily as their more fortunate neighbors.

Prior to the publication of the treatise on purgative medicines by the late Dr. Hamilton, Senior, of this city, there is much reason to believe that the use of laxatives was greatly neglected in such circumstances. But after the appearance of that work in 1818, an important reformation took place in this respect. Indeed, as in all important reforms in medicine, physicians and their patients soon ran to the opposite extreme; and ere long as much harm was done by the abuse of aperient and purgative medicines as previously by the neglect of them. At present it may be confidently said that both errors have been materially corrected. No one denies the great importance and frequent necessity of cathartics of all kinds, from the mildest laxatives up to the most drastic purgatives. And on the other hand, most physicians are now satisfied that gentle aperients are sufficient in numberless circumstances in which formerly powerful cathartics were the fashion. Among other observations, too, it has been found that the regular daily use, even of mild laxatives, is not so indispensable a precaution for preserving the health of those of a permanently costive habit as had been supposed by many physicians, and especially by many people themselves who were afflicted by that habit. For example, there can be no doubt, that for most people, who, though otherwise healthy, require constantly to use aperients, it is better to open the bowels in this way once every other day only, than daily by a daily dose. Some, especially those who live freely, require a more frequent dose. But in general you will find an effectual aperient every other day amply sufficient for those who do not augment the bulk of the alvine discharges by superfluous nourishment; and by that system they are much more likely to escape an irritable or congested state of the intestines arising, which we know to be the frequent consequence of the habitual excessive use of cathartics even of a mild kind.

Some persons, however, have such a horror of aperient medicines, that they cannot persuade themselves to take one oftener than twice a-week, or once a-week only. And, nevertheless, you will sometimes see them keep their health, and maintain their bodily comfort. But, for the most part, you will find it a sound general rule, to insist with such people on a more liberal use of aperients; and the great variety we now possess of convenient compound aperients, will enable you to find some one suitable to the constitution of any body, and reconcilable to almost any prejudices.

There are others whose prejudices are unconquerable, and who will not take laxatives at all, though their bowels do not move of themselves above once a-week, if even so often. And it is right you should be aware that this apparently most unnatural and preposterous habit is not of necessity, and in all cases, a habit injurious to health. You will often meet with men so singularly constituted, that they enjoy sound health upon a weekly stool. And, indeed, all perhaps that can well be said of them is, that they are rather to be envied by their fellow creatures, for an endowment which must be frequently found very convenient. But such people sometimes get into difficulties. About two years ago, a gentleman from Wigtonshire, a landed proprietor, attached to agricultural pursuits, and therefore never without free air and exercise, consulted me about a serious difference he had with his medical advisers in the country. Having recently recovered under their care from a severe pneumonia, they made the not unreasonable stipulation, when they ceased to attend him, that he should take a laxative every three days, to correct a constipated habit. To this he demurred, on the very natural ground, that, until his late illness, he had enjoyed excellent health for sixty years, although his bowels had been habitually moved all his life only once a fortnight. This gentleman had made a journey of 120 miles for no other reason than to get the question between him and his physicians settled by some competent authority in therapeutics; and, in referring to me for the purpose, he mentioned, for my further guidance, that a neighboring gentleman of his acquaintance, of the age of 70, had told him that he, too, had immemorably evacuated his bowels only every alternate Sunday, without being able to recollect having ever had an illness. It was scarcely to be wondered at that their common experience half inclined them to think that their constitution was the natural and patriarchal one.

Our hospital patient seems to have been of the same opinion with these elderly agriculturalists. Like them he has had some experience of life, being now 74. Like them, too, he has enjoyed singularly good health, being a surprisingly fresh-looking man for his years, notwithstanding that he had passed through severe trials in early life. As a soldier in India he sustained, when very young, a spear wound of the leg, where he has had, almost ever since, a small open ulcer, which he ascribes to the spear having been poisoned. In the Spanish war he was wounded at the battle of Barosso in 1811. There are now evident marks of the bullet having passed through him from the left groin, piercing the blade of the *os ilium* in its course. For two years he lay in hospital; and recovering with a shortened limb and stiff joint, he was invalided on a pension, as a wounded serjeant and soldier of twenty-one years' service. This he has enjoyed for forty-one years. Nor has his wound much incapacitated him; because for many years, and down to his present illness, he had actually worked as a railway laborer. During this long period he lived on his pension and wages in great comfort and sound health, until, on lately leaving off work, he became liable to constipation. At first his bowels were moved every other day in general, and afterwards seldom oftener than once a-week, unless he took physic, which he seldom did. At last the action of the bowels seemed to cease altogether, and he went four weeks without any evacuation, even though he made occasional trial of a laxative. At the end of the fourth week, a strong dose brought away a great accumulation. After that he had no further evacuation, and it is now three weeks ago. He had again made a few gentle attempts to assist nature; but he did not much insist upon this, because his lodging-house had no convenience, as he said, for a man under physic. During the entire period of seven weeks, he assures us he had no pain or other suffering whatever. But at last his belly got very large, so that his trousers would not button over it; and on this account he applied here for relief.

On admission he had no appearance of any suffering. He seemed a fresh, vigorous, active, cheerful man. He took his food tolerably well; the pulse was natural; and the tongue was only a little furred. "The abdomen," to quote the Hospital journal, "is much distended, especially in the iliac regions, where there are two large prominent swellings projecting laterally, so that the crest of the ilium on each side is quite sunk, the tumors projecting much beyond the bones. There are different irregular swellings at different parts of the abdomen, especially in the track of the colon. Over some of these points percussion is quite dull; over others it is tympanitic. The circumference of the abdomen, where largest, is 39½ inches."

As it was judged unsafe to give him active purgatives by the mouth at once, in case of the great gut being firmly obstructed with hardened fæces, a turpentine injection was properly administered by the clinical clerk in charge of him. The result was "a prodigious discharge of fæcal matter of all degrees of consistence," much of it composed of very hard scybala. A dose of jalap and calomel given immediately after this forerunner, brought away also a great mass of fæculent matter. Next day, being quite well, but with the abdomen as large as ever, another similar dose occasioned only an ordinary discharge. On the third day, the swelling being equally great, though now quite uniform, and everywhere clear on percussion, I gave him what has always appeared to me the most effectual of all safe energetic purgatives in cases of simple fæcal accumulation—two drachms of oil of turpentine with six drachms of castor oil in the form of emulsion. But he had only two scanty loose discharges, and the belly continued in the same state, presenting especially the singular enlargement and overlapping of the iliac regions.

It was now apparent that, owing to long continuous distension of the bowels with fæces and gases, their muscular coat had lost its tone, in some regions at least, and especially in the cœcum and descending colon. It was then proposed by the clinical clerk to resort to galvanism for relief from this paralytic condition, which suggestion was at once adopted. It is more than twenty-five years since galvanism was recommended as a useful remedy in cases of obstinate constipation; and we can easily see that it may be useful, and upon what principle it acts. The first way of using it was by directing the galvanic current from the mouth to the anus; and in that way it seems to have been most effectual and prompt in some cases. But its action is thus rather painful; and ulterior observation has shown that passing the current in various directions through the abdomen itself may be sufficient. This remedy seemed even more applicable to the state of our patient after the bowels had been cleared out. And accordingly it acted with wonderful energy and success. After the current had been passed for some time from before backward, as well as from side to side, he had in an hour a copious evacuation, in three hours another, and next morning a third. Flatus was also discharged in abundance; and the abdomen fell greatly, but still not completely, above all in the iliac regions. The pain of the galvanic action, however, had been so great that the patient begged to have a day's respite. In fact, he declared his willingness, and confirmed it with an oath, that he would rather be shot again than submit to be galvanized a second time. On the second morning, however, the remedy was applied more gently, and on two mornings subsequently. He had a daily discharge from his bowels, and sometimes two. The abdomen had now became natural in size and form. Since then he has had a natural evacuation every morning, without aid from either laxative or galvanism. He was dismissed after being fourteen days in the Hospital.

This is a case a little out of the common run, but not without instruction; and I have therefore thought it well to bring the chief circumstances under your notice. It is an excellent illustration of the influence exerted by galvanism over the animal functions. It appears to me to hold out a probability that the same remedy may prove servicable in restoring the tone of the intestinal muscles, in other forms of inconvenient chronic flatulent distension of the abdomen.—*Edinburgh Monthly Journal of Medicine.*

